# Active clearance vs conventional management of chest tubes after cardiac surgery: a randomized controlled study

**DOI:** 10.1186/s13019-021-01414-0

**Published:** 2021-03-23

**Authors:** Samuel St-Onge, Vincent Chauvette, Raphael Hamad, Denis Bouchard, Hugues Jeanmart, Yoan Lamarche, Louis P. Perrault, Philippe Demers

**Affiliations:** 1 Department of Surgery, Faculty of Medicine, Montreal Heart Institute, Université de Montréal, 5000 Belanger Street, Montreal, Quebec H1T1C8 Canada; 2 Department of Surgery, Faculty of Medicine, Hôpital du Sacré-Coeur de Montréal, Université de Montréal, Montreal, Quebec Canada

**Keywords:** Cardiac surgery, Critical care, Chest tube, Bleeding, Reexploration, Retained blood, Postoperative atrial fibrillation, ICU, Complications

## Abstract

**Background:**

Chest tubes are routinely used after cardiac surgery to evacuate shed mediastinal blood. Incomplete chest drainage due to chest tube clogging can lead to retained blood after cardiac surgery. This can include cardiac tamponade, hemothorax, bloody effusions and postoperative atrial fibrillation (POAF). Prior published non randomized studies have demonstrated that active tube clearance (ATC) of chest tubes can reduce retained blood complications prompting the ERAS Cardiac Society guidelines to recommend this modality.

**Objective:**

A randomized prospective trial to evaluate whether an ATC protocol aimed at improving chest tube patency without breaking the sterile field could efficiently reduce complications related to retained blood after cardiac surgery.

**Methods:**

This was a pragmatic, single-blinded, parallel randomized control trial held from November 2015 to June 2017 including a 30-day post index surgery follow-up. The setting was two academic centers affiliated with the *Université de Montréal* School of Medicine; the Montreal Heart Institute and the *Hôpital du Sacré-Coeur de Montréal*. Adult patients admitted for non-emergent coronary bypass grafting and/or valvular heart surgery through median sternotomy, in sinus rhythm for a minimum of 30 days prior to the surgical intervention were eligible for inclusion.

In the active tube clearance group (ATC), a 28F PleuraFlow device was positioned within the mediastinum. In the standard drainage group, a conventional chest tube (Teleflex Inc.) was used. Other chest tubes were left at the discretion of the operating surgeon.

**Results:**

A total of 520 adult patients undergoing cardiac surgery were randomized to receive either ATC (*n* = 257) or standard drainage (*n* = 263). ATC was associated with a 72% reduction in re-exploration for bleeding (5.7% vs 1.6%, *p* = .01) and an 89% reduction in complete chest tube occlusion (2% vs 19%, *p* = .01). There was an 18% reduction in POAF between the ATC and control group that was not statistically significant (31% vs 38%, *p* = .08).

**Conclusions and relevance:**

In this RCT, the implementation of active clearance of chest tubes reduced re-exploration and chest tube clogging in patients after cardiac surgery further supporting recommendations to consider this modality postoperatively.

**Trial registration:**

Clinical Trials NCT02808897. Retrospectively registered 22 June 2016.

**Supplementary Information:**

The online version contains supplementary material available at 10.1186/s13019-021-01414-0.

## Background

Shed mediastinal blood occurs to some degree in every patient undergoing cardiac surgery [1]. During the early hours of recovery, chest tubes positioned in the mediastinal, pericardial and pleural cavities are important to evacuate shed mediastinal blood and prevent retained blood around the heart and lungs. Chest tubes, however, are prone to clogging, which can impair drainage efficiency and engender complications [2]. Intrathoracic retention of shed blood may lead to cardiac tamponade requiring re-exploration or bloody effusions for which an invasive drainage procedure may become necessary [3–5]. Additionally, retained blood within the pericardium may promote both local proinflammatory and oxidative responses which are potent triggers for postoperative atrial fibrillation (POAF) and bloody effusions in susceptible individuals [6–9]. Meanwhile, although routinely used, traditional makeshift methods to clear visible clots like milking and stripping of tubes remain controversial as they present several practical and safety issues [10, 11].

To address these issues, recent prospective non-randomized studies sought to evaluate the impact of the implementation of a universal postoperative chest drainage protocol using active tube clearance (ATC) technology (PleuraFlow, ClearFlow Inc., Anaheim, CA). The role of this bedside drainage device, designed to provide superior chest tube patency by mechanically breaking clots within the lumen without compromising the patient’s safety or sterility, was acknowledged in the most recent guidelines for perioperative care in cardiac surgery by the ERAS Cardiac Society [12]. In recent observational studies, ATC was associated with lower rates of re-exploration for bleeding, POAF and fewer bloody effusions [13–16].

The present study is the first randomized controlled trial (RCT) designed to provide an assessment of the impact of ATC on complications associated with incomplete chest drainage and chest tube clogging after cardiac surgery.

## Methods

### Trial design and overview

The present study was an investigator-initiated, pragmatic, dual-center, parallel-group, single-blinded RCT conducted in Canada at the Montreal Heart Institute (MHI) and the *Hôpital du Sacré-Coeur de Montréal* (HSCM) over a three-year period. The trial was approved by both institutional ethics committee and conducted in accordance with the Declaration of Helsinki (2013). The study was registered on ClinicalTrials.gov (Registry number: NCT02808897). All patients provided written consent prior to enrollment.

### Study population

Adult patients undergoing non-emergent coronary bypass grafting (CABG) and/or valvular heart surgery through median sternotomy, in sinus rhythm for a minimum of 30 days prior to the surgical intervention were eligible for inclusion. Exclusion criteria included minimally invasive procedure, history of atrial fibrillation, presence of active endocarditis or myocarditis, admission for ventricular assist device implantation, heart transplant or transcatheter aortic valve replacement, cardiac surgical procedure requiring deep hypothermic circulatory arrest (≤18 °C), documented inherited bleeding disorder and allergy to the device material.

### Randomization

Eligible patients were randomly assigned in a 1:1 ratio, to either ATC or standard drainage protocol. Randomisation lists were generated by a biostatistician using the SAS PLAN procedure (SAS Institute, Cary, NC) and conserved on a secure server in each center. Randomisation was stratified by site and permutated blocks of sizes 4 and 8 were used. Enrollment was performed by trained clinical research staff. At the end of surgery, a member of the research team revealed the allocated randomization arm and accordingly delivered the allotted mediastinal chest tube in the operating room. Due to the nature of the treatment, only participants were blinded to the intervention.

### Endpoints

Prespecified endpoints included: 1) an episode of POAF ≥60 min on telemetry or electrocardiogram at any time between the index surgery through hospital discharge and 2) retained blood complications within 30 days post index surgery. The latter included the following interventions: re-exploration for bleeding or cardiac tamponade, pericardial drainage procedure – pericardiocentesis or pericardial window – or pleural drainage procedure – thoracentesis, pigtail catheter, new chest tube, thoracoscopy or lateral thoracotomy. A composite outcome for the total number of interventions in each group was also evaluated. Other relevant postoperative endpoints included transfusion requirements, in-hospital mortality, total length of stay and readmission within 30 days from index surgery.

As intraluminal tube clogging had never been compared between ATC and standard drainage devices in a large series, an exploratory visual evaluation of chest tubes upon removal was also performed. The first hundred patients to have their chest tubes removed whenever the investigator dedicated to the inspection was available, with no regard to their allocation, were selected for this sub-analysis.

### Data collection

Throughout the index hospitalization trained clinical research staff collected all data in case report forms and recorded them into a dedicated database. All patients were scheduled for clinical follow-up 1 month after surgery. At this time, research staff collected relevant details pertaining to the occurrence of adverse events that would have occurred since hospital discharge. Two blinded auditors independently reviewed primary and key secondary endpoints from every patient’s case report form comparing with information from the medical records. Discrepancy occurred in 6 cases (1.2%) for which the principal investigator reviewed the medical record in a blinded fashion to settle disagreements.

Direct visual inspection of chest tubes was performed by a single investigator. Upon removal, the chest tubes were cut transversely and classified as patent, partially or completely obstructed [2]. Pictures of every chest tube, minus the ATC device, which was removed when applicable, were submitted to a second blinded investigator. Both investigators agreed on all observations.

### Drainage strategy

All randomized patients received surgical, anesthetic and critical care management according to the center’s standard of care. Generally, at the end of cardiac surgery, the pericardium was left open and one (*n* = 413) or two chest (*n* = 107) tubes were placed in the mediastinum. If the pleura was opened during internal thoracic artery harvesting, a pleural drainage tube was added. Patients in the ATC group received one 28F mediastinal PleuraFlow device and conventional chest tubes for the remaining surgical sites (Clearflow Medical, Irvine, CA) [10]. For patients in the standard drainage group, conventional 28F polyvinylchloride chest tubes (Teleflex Inc., Morrisville, NC) were used. Chest tube positioning and use of 19F silastic Blake® drains (Ethicon Inc., Somerville, NJ) were left at the discretion of the operating surgeon.

The ICU nurses were instructed to use the ATC device by moving the external shuttle magnetically coupled to the inner clearance loop back and forth every 15 min for the first 8 h, every 30 min for the next 16 h, and finally every hour until removal of the drain as per the same protocol established in our institution since the initial clinical experience with this system back in 2011 [15, 17]. Milking and stripping of standard chest tubes were allowed in each group. If a severe intraluminal occlusion of a standard chest tube was suspected, direct aspiration was performed by a physician when deemed necessary. All ATC and standard chest tubes were connected to suction (− 20 cmH_2_O). Mediastinal chest tubes were removed 24 h after the index surgery or when drainage volume was less than 50 mL during the previous 8 h. Pleural and silastic drains were removed 48 h after the index surgery or when drainage volume was less than 150 mL during the past 24 h.

Details pertaining perioperative management of atrial fibrillation, antiplatelet therapy and monitoring of retained blood complications are presented as Supplementary methods.

### Sample size, power and statistical analysis

The sample size calculations were based on preliminary data from the participating institutions showing a local incidence of POAF of 25%. Prior observational data also suggested a 40% reduction in the incidence of POAF associated with the use of ATC. Using a X^2^ test to assess the primary endpoint, it was determined that a sample size of 254 patients in each arm was required to allow the detection of such an effect at a significance level of 0.05 with a power of 0.80. An interim analysis was performed after the first 300 procedures and the *p*-value was adjusted using the O’Brien-Fleming method.

Intention-to-treat analyses were performed for all primary and secondary endpoints. Categorical variables were compared using Pearson χ^2^ or Fisher exact test and presented as frequency and percentage. Continuous variables were presented as mean ± standard deviation or median with interquartile range (IQR) and compared using t or Mann-Whitney U test. A two-tailed *p*-value < 0.05 was considered statistically significant. All confidence intervals (CI) were estimated at the 95% confidence level. Analyses were performed using SPSS v. 25.0 (IBM Corp., Armonk, NY).

## Results

### Participants

**Fig. 1:**
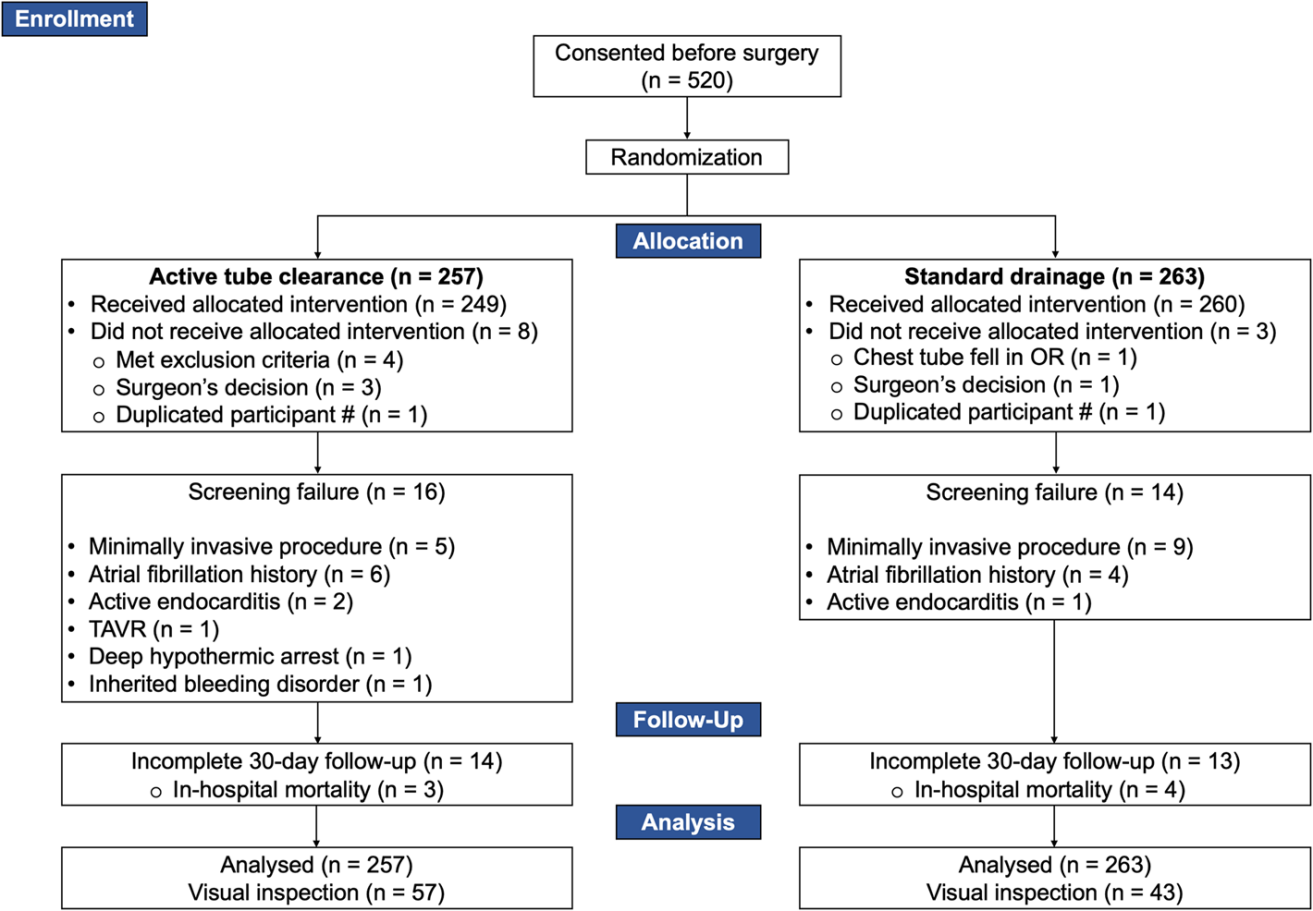
CONSORT Flow Diagram. A total of 520 patients consented to participate in this study before surgery and were randomized to receive either active tube clearance (*n* = 257) or standard chest drainage (*n* = 263). Of these patients, 11 did not receive the allocated intervention, for a cross-over rate of 2.1%. Thirty patients were included in the study despite being screening failures, mostly because of undergoing a minimally invasive procedure (*n* = 14) or presenting a history of atrial fibrillation (*n* = 10). Twenty-seven patients did not complete the 30-day follow-up period, for a completion rate of 95%. Of the 100 patients included in the visual inspection of chest tubes patency, 57 were allocated to ATC and 43, to standard drainage.OR, operating room; TAVR, transcatheter aortic valve replacement

From November 2015 to October 2018, 520 adult patients scheduled to undergo non-emergent cardiac surgery at the MHI or the HSCM were randomized to the ATC (*n* = 257) or standard drainage (*n* = 263) group. Eleven patients (2.1%) did not receive the proper allocation owing to reasons listed in Fig. 1. Thirty patients (5.8%) were randomized despite meeting at least one exclusion criteria. Data were recorded for all 520 patients, irrespective of protocol deviations, then analyzed according to a strict intention-to-treat principle. At 30 days, follow-up was 95% complete. Of the 100 patients included in the visual inspection of chest tubes patency, 57 were allocated to ATC and 43 to standard drainage.

### Baseline and operative characteristics

Baseline **(**Table 1**)** and operative characteristics were well balanced between groups. Mean age was 66 ± 10 years. Twenty-three percent (23%) of enrolled patients had complete cessation of antiplatelet therapy prior to surgery. Forty-one percent (41%) of patients were operated on an elective basis; 68% had isolated CABG, 26% underwent a valve procedure and 5% had concomitant aortic surgery (Table 2).

**Table 1:** Baseline characteristics

Variable	ATC (***n*** = 257)	Standard (***n*** = 263)
Age (yr)	66.6 ± 8.6	65.9 ± 10.8
Male	203 (79)	206 (78)
Body mass index (kg/m^2^)	30.0 ± 5.4	29.4 ± 5.6
LVEF	60.0 (50.0;60.0)	60.0 (50.0;65.0)
Arterial hypertension	208 (81)	213 (81)
Diabetes mellitus	113 (44)	97 (37)
Coronary artery disease	228 (87)	228 (89)
Peripheral arterial disease	48 (19)	38 (14)
COPD	48 (19)	42 (16)
Dyslipidemia	217 (84)	209 (80)
NYHA ≥ III	56 (22)	44 (17)
Unstable angina	60 (23)	69 (26)
Recent MI	78 (30)	78 (30)
Chronic kidney disease stage ≥3	31 (12)	39 (15)
Medication		
Aspirin	222 (86)	222 (84)
Warfarin	3 (1)	4 (2)
IV Heparin	84 (33)	94 (36)
Discontinued antiplatelet therapy	53 (21)	65 (25)
EuroSCORE II (%)	1.69 (1.00;2.73)	1.58 (1.11;2.82)

Variables are presented as *n* (%), mean ± SD or median (IQR)

*ATC* active tube clearance, *CCB* calcium channel blocker, *COPD* chronic obstructive pulmonary disease, *IQR* interquartile range, *LVEF* left ventricular ejection fraction, *MI* myocardial infarction, *NYHA* New York Heart Association, *PCI* percutaneous coronary intervention, *SD* standard deviation

**Table 2:** Operative characteristics

Variable	ATC (***n*** = 257)	Standard (***n*** = 263)
Elective status	101 (39)	110 (42)
Previous cardiac surgery	5 (2)	5 (2)
OPCAB	21 (8)	23 (9)
Circulatory arrest	3 (1)	2 (1)
CPB time (min)	69.0 (55.0;90.0)	67.0 (55.0;85.8)
Aortic crossclamp time (min)	50.0 (35.0:70.0)	46.0 (35.0;64.0)
Valve replacement or repair	70 (27)	63 (24)
Aortic valve	63 (25)	54 (21)
Mitral valve	7 (3)	9 (3)
Isolated CABG	174 (68)	182 (69)
CABG + Valve procedure	42 (16)	38 (14)
Aortic surgery	15 (6)	12 (5)
Ross procedure	1 (< 1)	5 (2)

Variables are presented as *n* (%) or median IQR

*ATC* active tube clearance, *CABG* coronary artery bypass graft, *CPB* cardiopulmonary bypass, *IQR* interquartile range, *IQR* interquartile range, *OPCAB* off-pump coronary bypass

### Postoperative complications

**Fig. 2:**
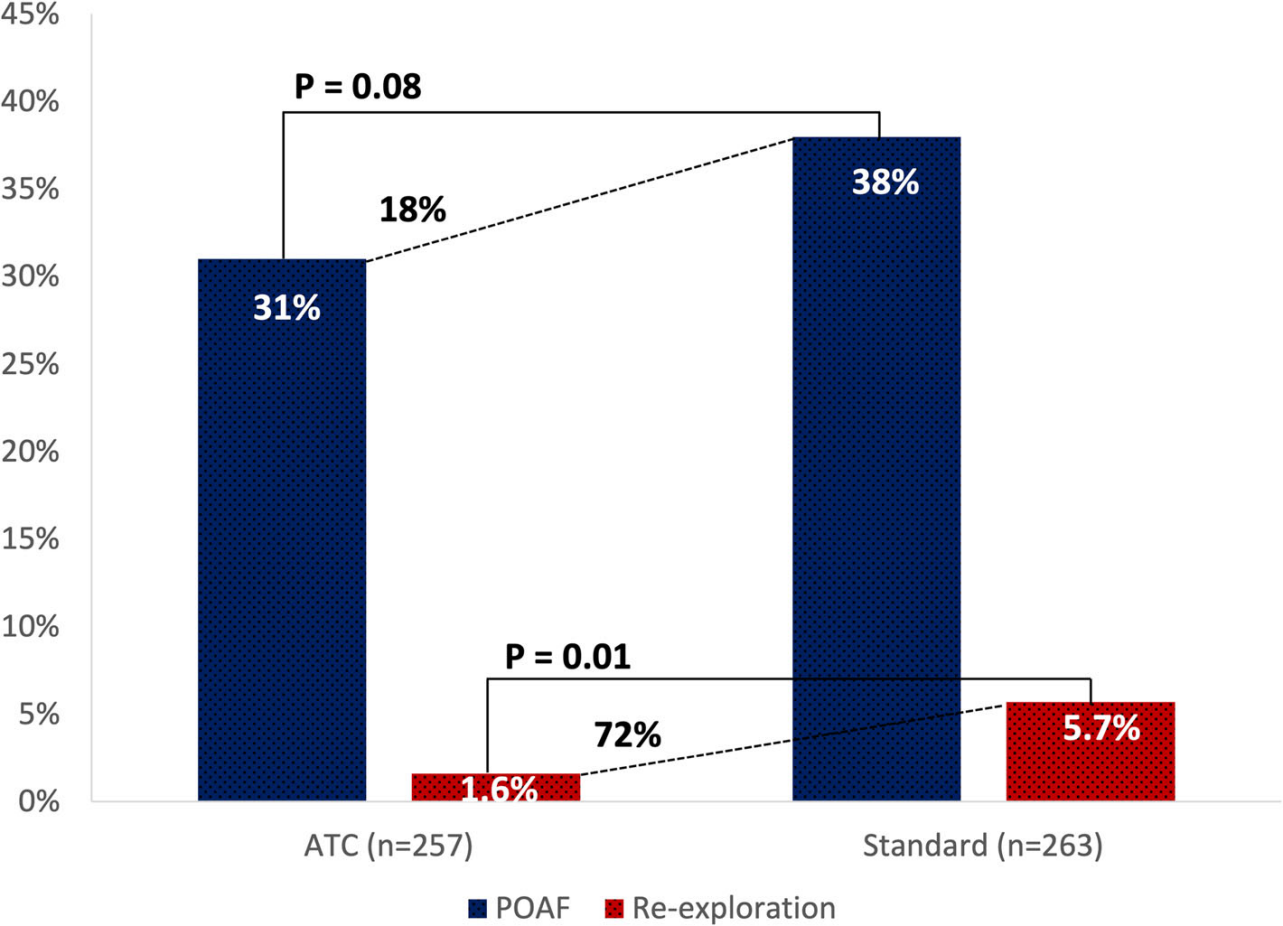
Active clearance of chest tubes decreased the incidence of postoperative atrial fibrillation by 18% (31% vs 38%, *p =* 0.08) and significantly reduced the rate of re-exploration for bleeding or tamponade by 72% (1.6% vs 5.7%, *p =* 0.01) when compared to standard drainage. ATC, active tube clearance; POAF, postoperative atrial fibrillation

ATC was associated with a significant reduction in the rate of re-exploration (1.6% vs 5.7%, *p* = 0.01), specifically for an indication of tamponade (0.4% vs 3.4%, *p* = 0.02) **(**Fig. 2**)**. This represents a relative risk reduction of 72% in the rate of re-exploration. Both surgical (1.2% vs 3.0%, *p* = 0.14) and coagulopathic (0.4% vs 2.7%, *p* = 0.07) bleeding were similar between groups **(**Supplemental Table 1**)**. The incidence of the composite outcome of total interventions for retained blood was similar between the 2 groups (6.2% vs 10.6%, *p* = 0.07). There was an 18% reduction in the incidence of POAF between the 2 groups (31% vs 38%, *p =* 0.08; Table 3), but this was not statistically significant.

**Table 3:** Postoperative endpoints

Variable	ATC (***n*** = 257)	Standard (***n*** = 263)	***p*** value
Atrial fibrillation	80 (31)	101 (38)	0.08
Retained blood complications			
Re-exploration	4 (1.6)	15 (5.7)	0.01
Bleeding	3 (1.2)	6 (2.3)	0.50
Tamponade	1 (0.4)	9 (3.4)	0.02
Pericardial intervention	3 (1.2)	5 (1.9)	0.73
Pleural intervention	9 (3.5)	8 (3.0)	0.75
Composite (total interventions)	16 (6.2)	28 (10.6)	0.07
Readmission			
Any reason	28 (12)	24 (10)	0.51
AF or retained blood complication	6 (3)	7 (3)	0.81
Postoperative allogeneic blood products	93 (36)	91 (35)	0.71
Red blood cells	79 (31)	75 (29)	0.58
Platelets	37 (14)	49 (19)	0.19
Fresh frozen plasma	7 (3)	15 (6)	0.09
Cryoprecipitate	2 (0.8)	11 (4.2)	0.01
Cardiac arrest	1 (< 1)	4 (2)	0.37
Stroke	4 (2)	4 (2)	>0.99
Acute renal failure	7 (3)	6 (2)	0.81
Sternal infection			
Superficial	8 (3)	8 (3)	0.95
Deep/Mediastinitis	10 (4)	4 (2)	0.09
Pneumonia	5 (2)	5 (2)	>0.99
Drainage for pneumothorax	10 (4)	8 (3)	0.60
Total ventilation time (h)	8.02 (6.60; 10.3)	7.97 (6.60; 10.8)	0.70
Total ICU time (h)	31.9 (22.2; 68.5)	27.0 (21.5; 66.9)	0.27
Hospital length of stay (d)	5.0 (4.0; 7.0)	5.0 (4.0; 7.0)	0.16
In-hospital mortality	3 (1)	4 (2)	>0.99

Variables are presented as *n* (%) or median (IQR). AF, atrial fibrillation; ATC, active tube clearance

### Visual inspection of chest tubes patency

Upon removal, visual inspection of chest tubes showed that ATC devices had a significantly lesser rate of complete obstruction compared to standard chest tubes positioned in the mediastinum (2% vs 19%, OR .08, 95% CI .01 - .65, *p* = 0.01). Standard chest tubes in other drainage sites showed similar obstruction patterns in both groups **(**Table 4**)**.

**Table 4:** Visual inspection of chest tubes

Variable	ATC (***n*** = 57)	Standard (***n*** = 43)	***p*** value
Mediastinal #1^a^ chest tube obstruction			0.01
None	42 (74)	26 (60)	
Partial	14 (25)	9 (21)	
Complete	1 (2)	8 (19)	
Mediastinal #2 chest tube obstruction			>0.99
None	7 (58)	4 (57)	
Partial	2 (17)	2 (29)	
Complete	3 (25)	1 (14)	
Right pleural chest tube obstruction			>0.99
None	11 (85)	17 (89)	
Partial	1 (8)	1 (5)	
Complete	1 (8)	1 (5)	
Left pleural chest tube obstruction			0.72
None	27 (82)	22 (88)	
Partial	6 (18)	3 (12)	
Complete	0 (0)	0 (0)	

Variables are presented as *n* (%)

*ATC* active tube clearance

^a^ Mediastinal #1 chest tube in the ATC group is always a PleuraFlow® device

### Other clinical endpoints

In-hospital mortality (1% vs 2%, *p*>0.99) and readmission (12% vs 10%, *p* = 0.51) were similar in both groups. ATC drainage protocol did not affect neither ICU time (31.9 [22.2; 68.5] hours vs 27.0 [21.5; 66.9] hours, *p* = 0.27) nor hospital length of stay (5.0 [4.0; 7.0] days vs 5.0 [4.0; 7.0] days, *p* = 0.16). Red blood cells, platelets and fresh frozen plasma transfusion rates were comparable between groups. Patients in the ATC group required significantly less cryoprecipitate transfusions (0.8% vs 4.2%, *p* = 0.01).

### Chest tubes data

Patients in both groups received the same number of drainage devices (2.29 ± 0.53 chest tubes vs 2.30 ± 0.57 chest tubes) (Supplemental Table 2**)**. Most patients (79%) had a single mediastinal chest tube (ATC = 77% vs standard drainage = 81%). No significant difference between groups was observed in regard to mediastinal or total chest tubes output (605.0 [430.0; 910.0] mL vs 650.0 [450.0; 977.5] mL, *p* = 0.26). There was no system malfunction with ATC devices throughout the trial period.

## Discussion

Chest tubes are required to facilitate pericardial evacuation of shed mediastinal blood and prevent hemodynamic collapse caused by cardiac tamponade. Chest tube output is also an important surrogate for postoperative bleeding and is thus monitored closely in the early postoperative hours. Maintenance of chest tube patency is thus of paramount importance as incomplete evacuation of shed mediastinal blood can have life-threatening consequences [3–5, 10].

**Fig. 3:**
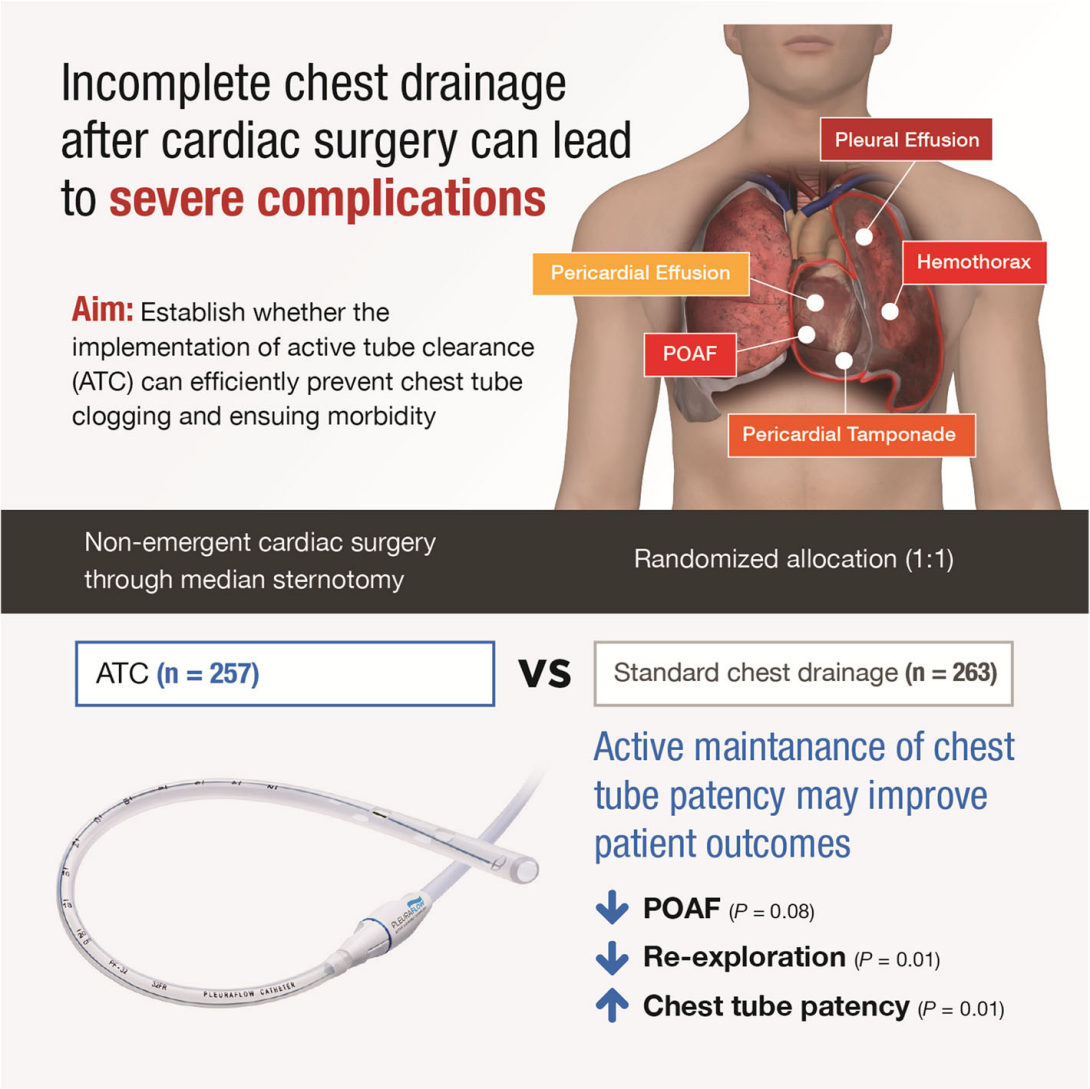
Graphical abstract. Chest tube clogging is common and can lead to retained blood around the heart and lungs. This study sought to evaluate the impact of a chest drainage protocol aimed at actively maintaining chest tube patency after cardiac surgery. Patients undergoing non-emergent cardiac surgery through median sternotomy were randomly allocated according to a 1:1 ratio to either ATC or standard drainage protocol. ATC was associated with non-significant reduction in POAF, lowered need for re-exploration for bleeding or tamponade and improved chest tube patency. This randomized controlled trial demonstrated that maintaining chest tube patency is paramount after cardiac surgery and may improve patient outcomes. ATC, active tube clearance; POAF, postoperative atrial fibrillation

In this trial (Fig. 3), the use of ATC was associated with significantly lower rates of reintervention due to bleeding or tamponade. Previous non-randomized studies have found similar results in patients undergoing left ventricular assist device implantation as well as in all-comers after cardiac surgery [14, 16]. These data suggests that actively maintaining chest tube patency with ATC may prevent tamponade by allowing more complete evacuation of shed mediastinal blood until bleeding ceases. There could be an additional effect as efficient decompression of the pericardium and provide a positive impact on the dynamic process of postoperative hemostasis by limiting fibrinolysis from retained clots. This mechanism may underlie the reduced need for re-exploration and cryoprecipitate transfusions observed in the present trial. The 72% RRR in the rate of re-exploration found in this study provides compelling arguments for the use of ATC given the morbidity associated with reoperation in patients in early recovery after cardiac surgery [5, 18–20].

Additionally, this is the first real-life proof-of-concept showing that ATC chest tubes exhibit higher patency than standard chest tubes. While the rate of partial obstruction, i.e. presence of non-obstructive clot either directly on the internal guidewire or in a crescent shape on the lumen surface, were similar between groups, the odds of complete obstruction impairing drainage was significantly lower in ATC devices than standard chest tubes. These results are consistent with the initial clinical experience held at our institution in 2011, in which standard chest tubes presented more obstructions compared to ATC, suggested by a lack of respiratory variation [17]. This finding is important as it demonstrates that ATC can safely diminish chest tube obstruction, deemed occurring in at least one-third of patients, especially by preventing clots at risk of significant clinical impact [2].

There is a growing body of literature linking chest tube clogging and retained blood around the heart in the triggering of POAF in susceptible individuals by promoting both local inflammatory and oxidative responses on the surface of the heart during early recovery [6–9, 21, 22]. The prospect of blunting this reaction by preventing blood from pooling around the heart with the simple addition of an ATC device is particularly appealing considering the steady incidence of POAF over the past decades despite pharmacological prophylaxis and the increased mortality and higher health care expenditure associated with this arrhythmia [23–25]. Although prior studies have shown a reduction in POAF in patients treated with ATC compared to those with standard chest tubes, this was not the case in this study. The sample size calculations performed prior to the study estimated a predicted reduction of 40% in POAF associated with ATC. Although this estimation was based on a retrospective study performed at our institution, this effect size may have been too optimistic and could have precluded the finding of significant results. In this study, the use of ATC resulted in an 18% relative reduction in the incidence of POAF, a number that most clinicians and surgeons would still find clinically relevant. Since previous observational studies have associated ATC with a reduction in POAF [15], it is possible that this trial was underpowered to detect the true effect of ATC. Thus, further studies are needed, powering for a lower RRR than 40%, to better assess this outcome in a properly statistically powered trial.

Although this study was not designed to evaluate healthcare economic benefits, a recent publication by Baribeau et al. in this journal demonstrated that patients treated with ATC reduced used of ICU resources and had mean hospitalization costs reduced by $2696. As detailed by Grieshaber and separately by Baribeau, although ATC costs more than conventional chest tube drainage, the costs are more than recuperated by the hospital purchasing department after the savings from complications avoidance [16, 26, 27]. This is an important consideration in our modern world of healthcare value where we strive to have the best possible outcomes at the least possible costs [27]. Our data supports this thesis of economic value for hospitals even after the purchase costs for ATC as well.

### Study limitations

In this trial, a number of factors may have limited the ability to detect the full effects of ATC. As previously mentioned, aiming to detect a 40% reduction in POAF may have been too optimistic. In many regards, the patients included in this trial were at low risk of post-operative bleeding. Expanding the eligibility criteria to include patients at higher risk of bleeding may have enabled to detect a greater impact of ATC. The use of multiple ATC devices could potentially have improved postoperative evacuation of blood from the pericardial sac and further reduce the incidence of POAF. However, since not all patients received pleural drains and, in an effort to make the treatment and control group as homogeneous as possible, it was decided to use only 1 mediastinal ATC device per patient. Finally, the use of additional mediastinal draining devices (eg: Blake tube) may be a confounding factor that needs to be taken into consideration when interpreting the results of this trial. These additional chest tubes were allowed since many surgeons in the participating institutions use both rigid and flexible (Blake drain) mediastinal drains.

## Conclusions

In this first RCT assessing the effects of a universal postoperative chest drainage protocol using ATC, the use of these devices was associated with lower rates of re-exploration for bleeding or tamponade and chest tube clogging. This supports the recent ERAS Cardiac Society recommendations that this modality should be considered for patients recovering from cardiac surgery.

## Supplementary Information

**Additional file 1.** Supplemental Methods.

**Additional file 2: Supplemental Table 1.** Surgical findings during the re-explorations for bleeding or tamponade.

**Additional file 3: Supplemental Table 2.** Chest tubes data.

## Data Availability

Data are available upon request.

## Abbreviations

ASA: Acetylsalicylic acid
ATC: Active tube clearance
CABG: Coronary artery bypass graft
CI: Confidence interval
HSCM: *Hôpital du Sacré-Cœur de Montréal*
ICU: Intensive care unit
IQR: Interquartile range
MHI: Montreal Heart Institute
POAF: Postoperative atrial fibrillation
POD: Postoperative day
RCT: Randomized controlled trial
RRR: Relative risk reduction
TTE: Transthoracic echocardiogram
