# Supplementary material for: Active clearance vs conventional management of chest tubes after cardiac surgery: a randomized controlled study
Source: J Cardiothorac Surg. 2021 Mar 23;16:44. doi: 10.1186/s13019-021-01414-0 (PMC7986555; doi:10.1186/s13019-021-01414-0)
Supplement: Supplementary file 1 — Additional file 1. Supplemental Methods. [file 13019_2021_1414_MOESM1_ESM.docx]

Supplemental Methods

**Atrial Fibrillation Management**

Monitoring of all patients by continuous telemetry from index surgery through hospital discharge is part of standard practice at both centers. According to our local protocol for prevention of POAF, β–blockade was used in all suitable cases and amiodarone was not given prophylactically to avoid potential adverse effects. Management therapy for a prolonged or symptomatic episode of POAF involved IV amiodarone, eventual conversion to oral administration when necessary, and incremental dose of β–blockade when appropriate.

**Perioperative Anticoagulation and Antiplatelet Therapy**

Acetylsalicylic acid (ASA) was discontinued at surgeon’s discretion, although never before CABG. P2Y12 inhibitors were withheld 5 (clopidogrel, ticagrelor) or 7 (prasugrel) days before surgery. Direct oral anticoagulants or vitamin K antagonists were discontinued 5 days preoperatively, without bridging. In all cases, appropriate medication was resumed or started according to a standard timeline: ASA on postoperative day (POD) 1, P2Y12 inhibitors and heparin on POD 2 and oral anticoagulants on POD 3.

**Monitoring of Bleeding and Retained Blood Complications**

Surgical re-exploration was performed when drainage volume, recorded hourly, consistently exceeded 200 mL/h despite optimized management of coagulation with rotational thromboelastometry [19]. Transesophageal or transthoracic echocardiogram (TTE) were performed on patients presenting hemodynamic instability to rule out hemodynamically significant pericardial effusion or tamponade. Whenever positive, pericardiocentesis or re-exploration was undertaken. Chest radiographs were routinely obtained preoperatively, upon chest tubes removal, and daily after surgery. Significant pleural effusions (˃500 mL), estimated by pulmonary sonography, were drained; smaller effusions were managed conservatively.
