# Supplementary material for: Active clearance vs conventional management of chest tubes after cardiac surgery: a randomized controlled study
Source: J Cardiothorac Surg. 2021 Mar 23;16:44. doi: 10.1186/s13019-021-01414-0 (PMC7986555; doi:10.1186/s13019-021-01414-0)
Supplement: Supplementary file 2 — Additional file 2: Supplemental Table 1. Surgical findings during the re-explorations for bleeding or tamponade. [file 13019_2021_1414_MOESM2_ESM.docx]

**Supplemental Table 1** Surgical findings during the re-explorations for bleeding or tamponade

| **Patient**  **Age (yr), gender** | **Group** | **Timing of reexploration (hours after index operation)** | **Indication for reexploration** | **Surgical findings** | **Categorization of the bleeding** |
| --- | --- | --- | --- | --- | --- |
| 74, male | ATC | 3 | Hemodynamic instability without overt tamponade on TEE | Diffuse non-compressive pericardial hematoma; oozing in regard of a bypass anastomosis | Surgical |
| 66, male | ATC | 5 | Pericardial tamponade on TTE | Diffuse pericardial hematoma; arterial bleeding from a branch of a right intercostal | Surgical |
| 66, male | ATC | 20 | Hemodynamic instability associated with right pleural hematoma on CT | Voluminous right pleural hematoma; no active bleeding | Coagulopathy |
| 73, female | ATC | 31 | Increased bleeding amount after Valsalva efforts | Absence of blood within the pericardium; oozing in regard of a graft anastomosis | Surgical |
| 68, male | Standard | 1 | Persistent significant bleeding | Diffuse non-compressive pericardial hematoma; no active bleeding | Coagulopathy |
| 75, male | Standard | 2 | Pericardial tamponnade on TTE | Diffuse pericardial hematoma; no active bleeding | Coagulopathy |
| 74, male | Standard | 4 | Pericardial tamponnade on TTE | Diffuse pericardial hematoma; active bleeding from a small branch of the left internal mammary pedicle | Surgical |
| 71, male | Standard | 5 | Persistent significant bleeding | Diffuse non-compressive pericardial hematoma with concomitant ipsilateral hemothorax; active bleeding from the left internal mammary pedicle | Surgical |
| 64, male | Standard | 7 | Pericardial tamponade on TEE and persistent significant bleeding | Localized pericardial hematoma compressing the right atrium; oozing in regard of transverse aortotomy line | Surgical |
| 75, male | Standard | 7 | Persistent significant bleeding | Diffuse non-compressive pericardial hematoma; arterial bleeding from a branch of a right intercostal | Surgical |
| 78, female | Standard | 8 | Pericardial tamponade on TEE | Localized pericardial hematoma compressing the right atrium; oozing in regard of transverse aortotomy line and canulation sites | Surgical |
| 64, male | Standard | 9 | Pericardial tamponade on TTE | Diffuse pericardial hematoma; significant bleeding from suture lines | Surgical |
| 69, male | Standard | 11 | Pericardial tamponade on TTE | Diffuse pericardial hematoma; arterial bleeding from a branch of a right intercostal | Surgical |
| 73, male | Standard | 16 | Persistent significant bleeding | Diffuse non-compressive pericardial hematoma; bleeding in regard of a bypass anastomosis | Surgical |
| 50, male | Standard | 22 | Persistent significant bleeding | Diffuse non-compressive pericardial hematoma; no active bleeding | Coagulopathy |
| 62, female | Standard | 26 | Pericardial tamponade on TTE | Localized pericardial hematoma compressing the right atrium; no active bleeding | Coagulopathy |
| 67, male | Standard | 44 | Pericardial tamponade on TEE | Localized pericardial hematoma compressing the right atrium; no active bleeding | Coagulopathy |
| 70, male | Standard | 69 | Pericardial tamponade on TTE | Diffuse pericardial hematoma; no active bleeding | Coagulopathy |
| 76, female | Standard | 140 | Hemodynamic instability without overt tamponade on TTE | Diffuse non-compressive pericardial hematoma; no active bleeding | Coagulopathy |

ATC, active tube clearance; CT, computed tomography; TEE, transesophageal echography; TTE, transthoracic echocardiogram
