# Supplementary material for: Active clearance vs conventional management of chest tubes after cardiac surgery: a randomized controlled study
Source: J Cardiothorac Surg. 2021 Mar 23;16:44. doi: 10.1186/s13019-021-01414-0 (PMC7986555; doi:10.1186/s13019-021-01414-0)
Supplement: Supplementary file 3 — Additional file 3: Supplemental Table 2. Chest tubes data. [file 13019_2021_1414_MOESM3_ESM.docx]

**Supplemental Table 2** Chest tubes data

| **Variable** | **ATC (*n*=257)** | **Standard (*n*=263)** | ***p* value** |
| --- | --- | --- | --- |
| Number of chest tubes |  |  | ̶ |
| 0 | 1 (<1) | 0 (0) |  |
| 1 | 4 (1) | 12 (5) |  |
| 2 | 173 (67) | 163 (62) |  |
| 3 | 77 (30) | 85 (32) |  |
| 4 | 2 (1) | 3 (1) |  |
| Chest tubes per patient | 2.29 ± 0.53 | 2.30 ± 0.57 | ̶ |
| Silastic drain | 14 (5) | 23 (9) | ̶ |
| Position of chest tubes |  |  |  |
| Mediastinal #1* | 257 (>99) | 263 (100) | ̶ |
| Mediastinal #2 | 58 (23) | 49 (19) | ̶ |
| Right pleura | 66 (26) | 80 (30) | ̶ |
| Left pleura | 207 (81) | 213 (81) | ̶ |
| Mediastinal chest tubes output (mL) | 405.0 (270.0; 600.0) | 450.0 (290.0; 657.5) | 0.09 |
| Total chest tubes output (mL) | 590.0 (420.0; 892.5) | 650.0 (430.0; 942.5) | 0.23 |

* Mediastinal #1 chest tube in the ATC group is always a PleuraFlow® device.

Variables are presented as n (%), mean ± SD or median (IQR). P-values are only presented for endpoints.

AF, atrial fibrillation; ATC, active tube clearance.
